# Enhancing the usability of health data for inequalities research: a UK quality improvement project and code list curation.

**DOI:** 10.23889/ijpds.v9i5.2687

**Published:** 2024-09-18

**Authors:** Tetyana Perchyk, Agnieszka Lemanska, Katriina L. Whitaker, Robert Kerrison

**Affiliations:** 1 University of Surrey; 2 Data Sciences Department, National Physical Laboratory

## Objective and Approach

Primary care datasets provide a rich source of information for inequalities research; however, the value derived from these depends largely on the comprehensiveness of the code lists used by researchers. To date, no standardised code list for inequalities research has been developed.

The aim of this project was to develop a comprehensive code list for groups of interest to inequalities research, namely: people with learning disabilities (LDs), people with severe mental illness (SMI), people from ethnic minority groups, and people who are transgender.

Existing code lists were extracted from the Clinical Practice Research Datalink (CPRD) Bibliography (the largest research dataset in the UK), and four UK code list repositories: OpenCodelists, Health Data Research UK, University of Cambridge, and London School of Hygiene and Tropical Medicine. Comprehensive code lists were then curated through collation and removal of duplicates.

## Results

16 code lists were identified for LDs, 18 for SMI, 16 for ethnicity, and 2 for transgender. From these, 661, 733, 346 and 68 unique codes were identified, respectively. Preliminary testing of the curated code lists, in a CPRD dataset, indicated that the number of individuals belonging to these groups was increased by 90% (compared to the original code list).

## Conclusions and Implications

Our findings suggest that the curated code lists improved data capture. These lists now need to be validated by healthcare specialists, before being made publicly available. The inclusion of code-specific definitions will enable international researchers to adapt the code lists to their medical systems.

